# How Can Primary Health Care System and Community-Based Participatory Research Be Complementary?

**Published:** 2010

**Authors:** Payam Sheikhattari, Farin Kamangar

**Affiliations:** 1 School of Community Health and Policy, Morgan State University, Baltimore, MD, USA

**Keywords:** Community, Research, Health, Iran

## Abstract

Health statistics leave little doubt that the current health system in Iran, which is mainly based on primary health care (PHC), is a functioning one, and that health in Iran has improved far beyond where it was 40 years ago. However, this system has its limitations too. While PHC is very effective in reducing morbidity and mortality from infectious diseases and other acute conditions, it is far less effective in addressing chronic and multifactorial conditions which are now emerging in Iran. In this article, we review some of the salient features of the current health system in Iran, its strengths and limitations, and then introduce community-based participatory research (CBPR) as a method that could potentially fill some of the gaps in the system. We will discuss the definition and steps needed to implement CBPR, provide some important references, and discuss how this approach may not only improve the health system but it could also lead to improvement in other fields in the society too.

## INTRODUCTION

Health statistics leave little doubt that the current health system in Iran, which is mainly based on primary health care (PHC), is a functioning one, and that health in Iran has improved far beyond where it was 40 years ago. However, the system has its limitations too. While PHC is very effective in reducing morbidity and mortality from infectious diseases and other acute conditions, it is far less effective in addressing chronic and multi-factorial conditions which are now emerging in Iran. In this article, we review some of the salient features of the current health system in Iran, its strengths and limitations, and then introduce community-based participatory research (CBPR) as a method that could potentially fill some of the gaps in the system. We will discuss the definition and steps needed to implement CBPR, provide some important references, and discuss how this approach may not only improve the health system but it could also lead to improvement in other fields in the society too. We hope that this article contributes to the discussions of the use of CBPR in the Iranian health system.

## THE CURRENT HEALTH SYSTEM IN IRAN

In a Lancet editorial,^1^ health systems in middle-eastern countries were described as adopting a “curative, rather than preventive” approach. But this is clearly not the case in Iran; Iran’s health system is mainly designed based on the model of PHC.

In 1978, all WHO members unanimously declared that access to basic health services was a fundamental human right, what was known as “Health for All by the Year 2000 (HFA 2000)”.^2^,^3^ PHC, which emphasized community-based preventive services, with substantial community involvement, was advocated as the main strategy to achieve the goals of HFA 2000.^2^,^3^ PHC entailed universal coverage of basic services such as safe water supply, promotion of food security, vaccination, family planning, education, control of endemic diseases, and provision of essential drugs.

Studies conducted more than 3 decades ago in Iran had already paved the way for PHC reform.^4^ However, the political and social changes of 1979, which almost coincided with HFA 2000 declaration, provided strong support for the implementation of PHC. The ideology behind HFA 2000 and PHC closely matched with the values of the time: social justice, equality, universal access to services, giving priority to the most vulnerable and underprivileged, and community involvement.^5^ PHC methods were carefully planned, and revolutionary fervor encouraged rapid implementation.

At the core of the Iran’s PHC plan was decentralization and empowering the rural areas with community health workers. Health houses were opened in 16,000 villages^6^ and were run by community health workers (behvarz). The behvarz were selected from the local community and were trained for two years to provide basic health services, including safe water, immunizations, and basic maternal and child care. Training methods involved group discussions, roleplaying exercises, and working in a model health house, rather than exhaustive memorization and other traditional pedagogical models.^5^ Being born and raised where they live and work, the behvarz typically have intimate relationships with their client community, are familiar with the norms of their society, and actively follow every person on basic health matters. To give an example of the effectiveness of this system, almost 100% of children born in Iranian rural areas receive BCG, diphtheria, pertussis and tetanus (DPT), polio, measles, and hepatitis B vaccines.^7^ Each health house on average covers four villages, and every few health houses are supervised by a “rural health center”. Iran has a total of 2300 rural health centers that are typically staffed by general practioners, dentists, midwives, pharmacists, nurse assistants, and other health workers.

In urban areas, the peripheral governmental health system generally starts from “urban health centers” that are similar in structure to rural health centers. However, in the very poor neighborhoods of larger cities, there are 600 “health posts” each manned by five health workers. Health posts provide PHC but not higher levels of care. Approximately 50,000 female volunteers aid the personnel of these health posts in pubic health education, family planning, child immunization, and other PHC priorities.^6^

Both Rural and urban health centers in each province are in turn supervised by medical universities, which have tertiary referral hospitals and medical facilities. Private practice offices and hospitals work in parallel and independently of the governmental system described above.

However, all private systems are also approved and monitored by the Ministry of Health. Private hospitals own < 7% of all 200,000 hospital beds, are located in larger cities, and provide services mainly to the more affluent urban population.

Public insurance plans provide almost free access to a variety of services offered in the governmental sector to approximately 90% of the urban and rural population. These services include tertiary referral procedures, such as coronary artery bypass grafts and renal transplants. Services offered in private practice offices are also covered by governmental insurance systems, but treatments in private hospitals are more expensive and usually require complementary insurance programs.

## HEALTH STATISTICS IN IRAN

Iran’s health statistics are close to the median of all countries in the world. Life expectancy is 71 to 72 years.^7^,^8^ For comparison, life expectancy in Turkey, Egypt and Pakistan are 72, 71, and 65 years, respectively. The infant mortality rate in Iran is 29/1,000, and corresponding rates in Turkey, Egypt and Pakistan are 21, 30, and 73 per 1,000 live births.^7^.

## THE EFFECTS OF IMPLEMENTING PHC IN IRAN

There is no doubt that Iran’s health status has significantly improved compared to 40 years ago, at least partly as a result of implementation of PHC. Infant mortality decreased from 164/1,000 live birth in the 1960 to 29/1,000 in 2007. During the same period, under-5 mortality rate decreased from 281 to 33/1,000, and life expectancy increased from 54 to 71 years.^7^

Improved health statistics are not unique to Iran. Except for a few countries which were struck by long-terms wars (e.g., Afghanistan) or acquired immune deficiency syndrome (AIDS) epidemics (e.g., Zambia and Zimbabwe), most countries have witnessed significant improvements in infant mortality rate and life expectancy. For example, in Pakistan, infant mortality rate decreased from 139/1000 in 1960 to 73/1,000 in 2003.^7^ Nevertheless, taking into consideration disruptive factors, such as political crises, the eight-year war with Iraq, and the low price of oil in the 1980s and 1990s, Iran’s progress in health has been considerable. Most experts believe that the establishment of health houses, employing the local behvarz, and political resolve to improve the basic health needs of the country, especially in rural areas, were fundamental to this progress.

The decline in mortality mainly reflects control of epidemics of communicable diseases, especially diarrhea and pulmonary diseases in children. However, with the decline of communicable diseases and progress in socioeconomic status, new diseases are emerging. Recent surveys have shown that 63% of the adult population (> 20 years) are either overweight (28.6%) or obese (14.2%).^9^ Cardiovascular disease, diabetes, and gastroesophageal reflux are becoming very common. Also recent cancer registries in remote areas of Iran have shown decreased rates of poverty-associated cancers (e.g., squamous cell esophageal cancer) and increased rates of affluence-associated cancers (e.g., breast and colon cancers).

## LIMITATIONS OF THE HEALTH SYSTEM IN IRAN

Despite the strengths of the current system, there is little doubt that it has limitations too. There are many countries that have far lower rates of infant mortality rate and higher life expectancies. For example, infant mortality rates are less than 10 / 1000 per year in approximately 50 countries. It is of note that lower infant mortality rates in Iran mainly reached a plateau after the mid 1990s. With higher life expectancy, chronic conditions such as heart diseases and cancers are becoming more prevalent. Since these conditions are multifactorial and affected mostly by lifestyle and multiple other factors, they are not easily remedied with PHC approaches. One way to reduce the gap between Iran and those countries is to purchase and implement extremely expensive equipments and facilities such as neonatal intensive care units but that is clearly not possible at the moment. Another way is to address the root causes at the community level through more efficiently using the currently available social and human capital. But how?

In Iran, people always look into the government to provide for their needs, and quite understandably so. When the entire budget and power is controlled by the government and all the decisions are made by the government, the community members do not feel ownership of the health system. Therefore, they are not willing to participate and use their own resources to improve the status quo. But we believe, citizens are highly capable of providing assistance, becoming partners, and improving the system. Consider the following two examples.

Example 1. Reducing maternal mortality rate requires community awareness about the signs and symptoms of high risk pregnancies and immediate action in terms of transportation and medical care. Emergency services are usually not accessible in rural areas. However, a participatory project called “Alarm System”, conducted in Kurdistan Province from 2000 to 2002, mobilized the community to actively identify and transport at risk pregnant mothers to the health system, which led to significant reduction of cases of maternal mortality. This project reduced the annual number of pregnancy related mortality in the area of the project from 16 cases to 7 (unpublished data).

Example 2. The PHC currently practiced in Iran is to some extent a onesize-fits-all system. The services provided in different parts of the country are very similar. However, if the community feels that they have issues that are not addressed adequately, e.g., a need for emergency obstetrics services, those needs should be discussed and approved at the city and province health departments, which may take a long time or never happen. The community may be quite competent in creating and sustaining such services, if approached and consulted through an equal partnership.

## COMMUNITY-BASED PARTICIPATORY RESEARCH

In the United States, health professionals usually refer to the health clinics, hospitals, doctors, and nurses as the major players in the health system. Iranian health experts, like other professionals all around the world, usually describe the network of the healthcare institutions starting from the Ministry of Health & Medical Education and extended to remote villages through PHC as the Iranian health system. However, “health system” can be much more extensive than that, for example, by involving the households,^10^,^11^ letting them identify their own problems and contribute to ways of solving them. An analogy would be the agricultural system.

If you ask agricultural experts to describe the national agricultural system, they would not limit the system to the Ministry of Agriculture, its offices, and experts. They are more likely to describe lands, crops, and farmers as the major players. The point is that in the agricultural system the definition is based on the crops production and it emphasizes the farmers’ role as major producers. With a similar mindset, why should we limit the national health system to hospitals, clinics, and health centers and health houses? Why not engage mothers and other community stakeholders within the society for producing health like the role farmers play at the agricultural system?

One of the promising approaches to address this issue has been developed under the rubric of *Community-Based Participatory Research* (CBPR).^12^ CBPR is defined as a “Collaborative approach to research that equitably involves all partners in the research process and recognizes the unique strengths that each brings”.^13^ We would like to emphasize, however, that although CBPR entails the word “research’, it is not solely for the purpose of research and increasing knowledge. Research in CBPR is a strategy to achieve the broader goal of social change which serves as the main incentive for community members to partner with the health system. Through CBPR, healthcare institutions and community-based organizations team up to identify the needs and to build on community strengths in order to address them. Healthcare experts and community stakeholders will share their technical and experiential knowledge and learn from each other. They also share power and produce a climate of equal participation in every step of program design, implementation, and evaluation.^12^,^14^

CBPR is more than a “minimal” involvement of the community. Many strategies used to involve the community have been centered on the opinions of the health experts and have followed a top-down approach. For example, many health clinics still view clients as passive consumers of professional opinion. Such approaches are defined in the literature as expert-driven initiatives that entail minimal involvement of community members in designing and implementing health interventions.^15^ This mind-set presumes that behavioral problems result from people’s lack of knowledge^16^ and the expert’s role in problem identification and program design are sufficient for solving the problems. These authoritarian approaches may meet resistance from the community,^17^ and may not lead to the development of the community. In contrast, community development approaches strive to avoid such top-down views to both problem identification and intervention design.^15^ CBPR, for example, relies on the strengths of local communities, including the skills and assets of individuals, as well as their networks of relationships, to build trust and create mutual commitments.^18^

## CBPR PRINCIPLES

CBPR is a philosophical approach that can be applied to a wide variety of situations, rather than solely to research. For example, if epidemiological studies are designed and implemented in partnership with the community, rather than being driven mainly by experts, they will become CBPR studies. Likewise, health system can practice CBPR, if health clinics build equal partnership with the local community and collaboratively identify the priorities and build on local assets. In both cases, CBPR is about making equal partnership with the community to better achieve the health outcomes.^12^

Being open to *learning* and *capacity building* are two main principles of CBPR^12^. Health professionals and community members team up to learn from each other and support each others’ activities. Health experts will learn how the “real” world works and why; community partners will learn what evidence-based healthcare requires and why. Capacity building is about increasing the assets and resources. They can include budget, equipments, facilities and even more important human capital. Learning enhances the human competencies and empowers both individuals and institutions.

In community partnerships, it is important to build on community strengths, and not emphasize weaknesses. For example, if the community has a school or a mosque, discussions around such “assets and strengths” may initiate positive and innovative thinking on how to use them to address health problems. In contrast, focusing on negative issues, such as weaknesses, may lead to a counter-productive environment in which the discussants may look for people who should be blamed for the deficiencies, which in turn will lead to defensive behaviors among others. It is also important to base the discussions on evidence-based practices, rather than local unproven/harmful traditions, to mitigate un-productive tensions.

As mentioned earlier, CBPR is based on equal partnership, which relies on equal access to and control over resources, such as budget, knowledge, data, etc. Unequal partnership will be detrimental to equitable participation. For example, if the budget is controlled by one partner, the other partner’s role becomes more advisory than equal. Another situation is when experts don’t share technical knowledge, necessary for an equal partnership, with the community. Someone may argue that experts are not able to communicate their technical expertise acquired through many years of education to those who lack a similar background. That is true, and we don’t mean that within a partnership different players should become alike or change places. Health experts need community because they may know better about their own problems and they have better access to local resources. On the other hand, community needs health experts because they are the ones who have the technical knowledge on how to address the health problems. This relationship works best when each party brings their own strengths to the table, and this requires effective communication. Therefore, it is necessary to translate technical terms into a plain language to create a common understanding of the issues. An informed community, with adequate knowledge about the effectiveness of health interventions as well as their potential consequences, will be able to come up with innovative and locally appropriate solutions.^19^

## PRACTICAL STEPS FOR DESIGNING AND IMPLEMENTING CBPR PROJECTS

CBPR involves several steps, including defining the community, building and sustaining a relationship with the community, and establishing the rules of engagement.

### 

#### Defining the community:

In CBPR, community is defined as a unit of identity and may refer to membership in a family, social network, geographic neighborhood, and/or other socially created dimensions of identity.^12^,^20^ The definition of the community depends on the nature of the problem, available resources, and environmental characteristics and that may include not only people who receive services but also organizations that provide services. For example, the health system may define the community by ethnic background and geography (e.g., Turkmens in Eastern part of the Golestan Province), then based on the nature of health program (e.g., esophageal cancer prevention). The definition may be narrowed down to different subgroups and community stakeholders (e.g., individuals 30 years of age of above and internists in the area). Definition of the community is as important as defining the goals and objectives of the program and it is more effectively accomplished in collaboration with potential partners. When community is defined in appropriate terms, program administrators are able to look for key influential members that may represent the defined community. Influential members of each community usually play their roles through membership in local groups/institutions. Snow-balling is a useful technique for inquiring further information from the existing informants in order to learn more about other key players of each community.^21^,^22^ An example of snowballing is when we ask each key person to identify and enroll 2-5 other active players. Participation in CBPR is about making relationship and partnership with the local community.^23^ Therefore, it is important to carefully look for incentives and mutual benefits that may bind the health system and community in a sustainable and mutually beneficial manner.

#### Building and sustaining the relationship:

Relationship building is an iterative and time-consuming process and should be regarded as an investment. While it may not lead to quick results, these relationships have the potential to last much longer and allow for creativity in developing networks, resources and sustainability.^24^ It starts with meeting with and carefully listening to the influential members of the community. Like any other partnership, health system and community should negotiate their expected gains from the relationship. It becomes particularly challenging, if one partner is not flexible enough to change and meet the needs of the others. Memorandum of Understanding (MOU) is a way to formalize the relationship into an agreement with clear terms and conditions.^25^ A well prepared MOU entails perceived expectations and responsibilities of all partners. Also, it includes rules and regulations to ensure an equitable partnership that balances research and action for mutual benefit of all partners^25^. For example, it is very important to make it clear how resources are accessed and controlled (e.g., budgets, equipment, facilities, data, etc.) and how the power is balanced to ensure an equal partnership. Again community connection takes time but, if successful, it will yield invaluable results and will develop and sustain over time. International Committee of the Red Cross is a successful model of partnership between non-for-profit organizations and governments that has sustained and evolved into a large global organization over time.^26^ Within the health system small urban and rural health clinics have the potential to become many times more effective and less costly, if they connect to community and make strong partnerships.

#### Establishing the rules of engagement:

Building a new partnership is similar to creating a new institution. Partners come to the meetings with different visions- sometimes contradictory- and it is very important to create a receptive environment that acknowledges differences and fosters constructive negotiation.^27^ The rules of engagement are about values, procedures, and a shared vision that everyone accepts. The partners may come up with a list of behaviors that are permitted as well as behaviors that are not. Solving emerging problems will become a challenging task, if partners do not make decisions at the beginning of the partnership on how to resolve conflicts.^28^ For example, they may decide to make decisions through consensus or majority vote in different situations. MOUs usually have information about different structures for the partnership. For example, Community Advisory Board (CAB) has been defined in many CBPR projects as the main community-based structure for decision making.^25^ However, it is essential to define the rules of engagement for the CAB early on the process in terms of the frequency of the meetings, groups dynamic procedures (i.e., facilitation, note taking, problem solving, etc.), and deliverables. CAB may envision potential working groups or sub-committees in the first few meetings that help the partnership to get better organized.

## SOME COMMONLY USED METHODS IN CBPR

**Figure 1:**
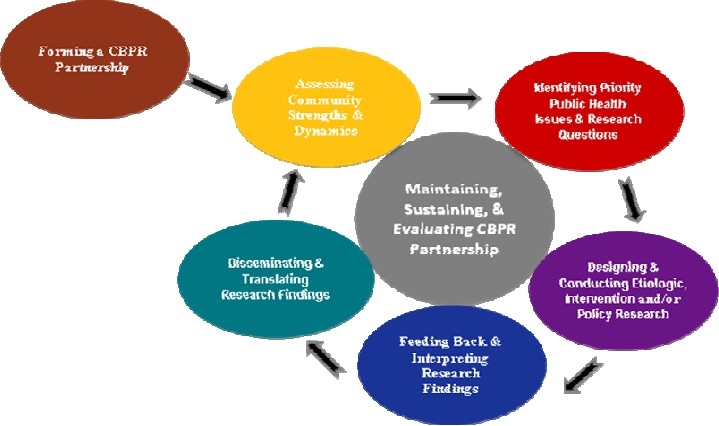
Core Components/Phases in Conducting Community-based Participatory Research (CBPR)

CBPR projects follow the traditional cycle of program design, implementation and evaluation. Forming a CBPR partnership is the first step that needs to be taken before entering into the programming cycle (Figure 1). Most of the partnerships start with a small group of partners representing the community and the health system. The core members of the partnership will take actions to expand the partnership through recruiting new members and creating more structures. Community and academic partners play different roles in a CBPR project. Community members and organizations may serve as key informants about the community, its assets, resources, goals and vision. They may contribute to designing research instruments and/or culturally relevant interventions. In addition, they may help to recruit clients and serve as messengers to disseminate health information. Academic partners, on the other hand, can serve as managers, leaders, technicians, or consultants in different projects.^13^ Partnership with the community is not all about engaging community members in the health system’s activities. CBPR is a two-way relationship between the local health system and other local members/institutions. For example, contributions of a local teacher in a health clinic should open doors for a systematic and mutually beneficial relationship between the local health system and the local school system. In an equal partnership, everybody’s input is important even if some of the ideas seem contradictory. Successful partners are open to new ideas and constructively engage in critical analysis of the options without taking sides. Health system has to realize that valuing other opinions requires giving up some of their power and letting new ideas find their ways in changing the old traditions.

In a new partnership, identification of community needs and assets and selection of the priority issues are initial steps.^29^ Different methods have been introduced in the literature for community needs assessment and assets mapping.^30^ Methods such as participatory rural appraisal (PRA), rapid rural appraisal (RRA), and street intercept surveys (SIS), as well as qualitative methods such as focus groups discussions (FGD) are extensively used in CBPR projects.^30^–^33^ These methods provide easy-to-implement tools for community needs assessment and identification of the assets that can be utilized by leaders and community members with minimum research experience and education. Partners identify the real needs of the community within the context of available local assets and resources. Local data collection is an important phase at every level of the programming cycle. The process requires initial negotiation between the partners about the needed data for the project as well as planning for data collection and analysis activities. Data collection activities will lead to creation of learning organization, in which major players actively seek evidence and learn from their experiences.

## CREATING A LEARNING ENVIRONMENT

In traditional research, community is perceived as an object of research. It happens often that although the results of research are disseminated in national and international conferences and journals, the community may not learn about the results and their implications for a long while, or at all. In contrast, in CBPR, research results and their application are integrated; since the community is a partner from the beginning to the end, they will see the results and may help in instant implementation. In order to achieve this goal effectively, a learning environment needs to be created. A learning environment needs a shared vision, members familiar with easy-to-implement data collection methods, and a system of implementation with accountability.

Learning environments require participation of all key stakeholders in designing a future they collectively desire, called their “shared vision”, and realizing that future through collective action.^34^ Nurturing a learning environment according to the shared vision creates an effective strategy to deal with contemporary growing health problems, such as obesity, which cannot be solved with simple solutions used in the old models. Fostering a shared vision aligns the efforts of the stakeholders in solving the problems, which is very important in utilizing human ingenuity and capacity to lead. This vision provides direction for the partners to focus on making communities and households more resourceful and doing the right thing. Also, it is important to bear in mind that for doing the right thing they should learn about the facts through collective leadership and using reliable, valid and practical research methodologies.^30^

A learning environment encourages every member to continuously acquire valid and reliable evidence and come up with locally appropriate interventions that improve the performance of the primary producers of health. Data collection and interpretation help local partners to improve the quality and capacity of their local institutions and turn them into learning organizations that actively seek innovative ways for higher achievement. Research at the community and PHC clinic levels is usually perceived as a highly professional and hard-to-achieve task requiring academic skills and resources. How-ever, there are new qualitative and quantitative research skills that local managers and community members can acquire with some short term training.^19^ Therefore, they will be able to design and implement local inquiries and use the findings to address the local health problems.

Leadership and management are both necessary processes in creating learning environments through implementation of the interventions with accountability. Setting the direction of the change is fundamental to leadership, and management helps system to properly work.^29^,^34^ Both leadership and management need valid and reliable information regarding the local settings and can ensure the availability of such data through doing proper research, and making and using local level measurements. However, learning is an essential process at all levels and requires challenging the old mental models when encountering new evidence.^28^This happens only when we have an open mind, and temper our speaking with the art of listening, when we are both creative and open to the others creativity. This culture should be created from inside mainly by local leaders who willingly engage stakeholders, assure creativity and produce new things through nurturing a learning environment. Partners in these kinds of relationship can be accountable for the gains and take credit for their achievements.

## SUMMARY AND RECOMMENDATIONS

While PHC has contributed to reducing mortality and improving health in Iran, it might have reached its limits. Incorporating CBPR may further improve health. CBPR provides an interesting orientation to both research and healthcare services. It is not, however, a specific method. Community participation is proven to be important to achieve equity in health and requires equal and balanced partnerships among members. PHC health clinics are usually funded through public and private sources and are required to report their health activities and the outcome of the projects to the funding agencies. Lack of partnership with local community may create a situation that projects are designed, implemented, and evaluated by the experts without taking into consideration the local context and the perceived needs of the local actors. In addition, such a top-down model usually is not able to identify and take advantage of the important community assets. Community’s lack of engagement in the local health system makes the health services more expensive and less sustainable due to its high dependence on the external funding sources. For example, there are PHC clinics that have to request everything they need from the city health department. However, many of those requests could be taken care of with minimum cost at the local level (e.g., replacing a broken window).

A well-structured health center that partners with the community, which we call a community health center (CHC), makes a working relationship with the influential members and/or institutions from its catchment area. The relationship is win-win and follows the simple principle of helping others in order to be helped. CBPR provides a step-by-step framework on how to partner with the community and how to expand and sustain this partnership. A CHC benefits from different types of resources within the community such as human capital, physical resources, and the social network. Also, community institutions get support for their projects and find the CHC a great asset for improving health and wellness of their community. The question is: what steps are necessary for PHC clinics in order to become a well-structured CHC?

The PHC clinic’s mission is very close to the CHC’s mission. They both want to improve the health and well-being of the community through health promotion and disease prevention. A PHC clinic, however, needs to take some additional steps in order to become fully connected to the community and call itself a well-structured CHC. First, partnership with local community requires some degree of autonomy and flexibility of the funding.^35^ It is not possible to form an equal partnership if the community demands some changes and the health providers keep saying that they cannot oblige. PHC clinics must have control over some of the critical resources such as funding, materials, and equipments. Also, the clinics should be flexible to some degree in terms of setting the priorities and planning specific interventions. In many health systems, this condition can be ensured through making national or regional policies as part of a healthcare reform initiative. The second requirement is the provision of short-term training on CBPR and appropriate local data collection techniques to the staff of the PHC clinics.^19^ In the United States, CBPR is one of the 8 new areas in which schools of public health should emphasize in their curricula.^36^ Participation of potential partners will improve the quality of the training and may clarify some of the planning steps that are needed in each setting. And third, building a partnership can be achieved in many different ways and may yield various results depending on the characteristics of the local setting. Health system will benefit substantially from creating an environment for documenting and sharing best practices and lessons learned from different partnerships.

We fully recognize that creating partnership in health and using CBPR is a slow process and requires years of work. However, if people become partners and learn to solve their own problems, the effects will not be limited to health. This will be a vision and approach that the community can take to address a large number of its other problems too, and therefore in the long run it may lead to substantial improvements in many aspects of the life of the society.
